# Energy reserves and accumulation of metals in the ground beetle *Pterostichus oblongopunctatus* from two metal-polluted gradients

**DOI:** 10.1007/s11356-012-0993-y

**Published:** 2012-06-06

**Authors:** Agnieszka J. Bednarska, Izabela Stachowicz, Ligia Kuriańska

**Affiliations:** Institute of Environmental Sciences, Jagiellonian University, Gronostajowa 7, 30-387 Kraków, Poland

**Keywords:** Lipids, Carbohydrates, Proteins, Zinc, Cadmium, Carabids

## Abstract

Living in an area chronically polluted with metals is usually associated with changes in the energy distribution in organisms due to increased energy expenses associated with detoxification and excretion processes. These expenses may be reflected in the available energy resources, such as lipids, carbohydrates, and proteins. In this context, the energy status of *Pterostichus oblongopunctatus* (Coleoptera: Carabidae) was studied in two metal pollution gradients near Olkusz and Miateczko Śląskie in southern Poland. Both regions are rich in metal ores, and the two largest Polish zinc smelters have been operating there since the 1970s. Beetles were collected from five sites at each gradient. Zinc and cadmium concentrations were measured in both the soil and the beetles. The possible reduction in energy reserves as a cost of detoxifying assimilated metals was evaluated biochemically by determining the total lipid, carbohydrates, and protein contents. At the most polluted sites, the Zn concentration in the soil organic layer reached 2,906 mg/kg, and the Cd concentration reached 55 mg/kg. Body Zn and Cd concentrations increased with increasing soil Zn and Cd concentrations (*p* = 0.003 and *p* = 0.0001, respectively). However, no relationship between pollution level and energetic reserves was found. The results suggest that populations of *P. oblongopunctatus* inhabiting highly metal-polluted sites are able to survive without any serious impact on their energy reserves, though they obviously have to cope with elevated body metal concentrations.

## Introduction

Metal-contaminated areas are found all over the world due to atmospheric deposition from smelters and other metallurgic processes, and the most contaminated areas are usually located up to several kilometers from the emission source. Metal pollution may have a drastic impact on ecosystems, and several studies have shown a reduced density of soil invertebrates in the vicinity of the emission source (e.g., Spurgeon and Hopkin 1999). On the other hand, some invertebrates can tolerate metal concentrations far above levels known to induce lethal effects in non-tolerant populations (Stürzenbaum et al. 1998). Viable populations of animals have been found at soil contamination levels much higher than the critical concentrations of metals established for soil or food in the laboratory (Posthuma and Van Straalen 1993). Such inconsistencies show that results derived from laboratory experiments cannot be extrapolated easily to expected field effects, and studies on field populations are crucial and necessary.

Living in an area chronically polluted with metals is usually associated with changes in the energy distribution of organisms due to increased energy expenses associated with detoxification processes (Sibly and Calow 1989). However, the energetic budget of an organism is restricted. The major share of energy is used for maintenance and only the surplus can be devoted to biomass production via growth and reproduction. Maintenance requirements increase in organisms living in polluted environments, resulting in a reduction of energy for growth and reproduction (Sibly and Calow 1989). Therefore, the total amount of energy available for maintenance, growth, and reproduction may provide a sensitive measure of stress in an organism. Although the significance of detoxification costs in an organism’s energy budget and their effects on fitness has been the subject of many studies (Khalil et al. 1995; Laskowski et al. 1998), such costs were found directly in only a few studies (Rowe et al. 1998; Hopkins et al. 1999; Pook et al. 2009). Some indirect proof is available regarding the costs incurred by toxicant-resistant individuals, such as lower reproduction (Łagisz et al. 2002) or decreased tolerance to other stressors (Stone et al. 2001).

The difference between available energy reserves (based on the biochemical analysis of carbohydrates, lipids, and proteins) and energy consumption has been shown to be indicative of an organism’s overall condition (De Coen and Janssen 2003a), and a decrease in the available energy reserves can be used as a biomarker of metal stress (Scott-Fordsmand and Weeks 2000). The majority of studies on energy budgets have been performed with aquatic organisms (De Coen and Janssen 1997; 2003a; 2003b; Verslycke et al. 2004a; Erk et al. 2008; Smolders et al. 2003). Reduced energy reserves were reported in a study of protein, glycogen, and lipid contents in isopods from terrestrial ecosystems (Donker 1992). Maryański et al. (2002) observed decreased energy reserves in beetles exposed to Zn- or Cd-contaminated food in the laboratory.

In terrestrial ecosystems, carabid beetles are one of the invertebrates most resistant to metals, and many carabid species inhabit highly contaminated areas (Skalski et al. 2011). Beetles may acquire metals from soil through dermal absorption (larvae) and feeding on contaminated food (larvae and adults), and as a species with low dispersal ability (Brunsting 1981) may be confined to local zones of pollution for multiple generations. At the same time, these beetles are poor metal accumulators (Laskowski and Maryański 1993; Butovsky 2011), which may be the result of efficient detoxification and excretion mechanisms (Janssen 1991; Kramarz 1999). However, individuals inhabiting chronically polluted environments appear to be less tolerant of additional environmental stressors, such as pesticide treatment or food deprivation, than individuals from uncontaminated areas (Stone et al. 2001). This observation is indirect evidence of the costs of living in a polluted environment, which may result from a restricted energy budget when the allocation of energy to stress resistance increases survival but leaves less energy for other processes. Thus, the cost of metal tolerance can be observed as reduced energy reserves in populations from polluted environments compared to reference populations. Because the response of an animal to stress is a complex reaction, which depends not only on the type of chemical but also the many internal and external factors, studying two or more gradients, each with a number of differently polluted sites, allows for more general conclusions and provides better control over possible confounding factors. In the present study, energy reserves and body metal concentrations were determined in field-collected ground beetles, *Pterostichus oblongopunctatus* (Coleoptera: Carabidae), in order to investigate the costs associated with inhabiting areas chronically polluted with metals.

## Materials and methods

### Test organism

The ground beetle *P. oblongopunctatus* (Coleoptera: Carabidae) is a widely distributed species in European woods and can be considered a typical representative of epigeic carnivorous insects. As a non-specialized carnivore, this beetle may potentially be exposed to elevated concentrations of toxins in its diet. The generation time is generally 1 year, though a small proportion of adults may live up to 3 years (Brunsting 1981).

### Research area and beetle sampling

Adult beetles were collected from sites located along two metal pollution gradients, in the vicinity of zinc and lead smelters near Olkusz and Miasteczko Śląskie (gradients labeled OLK and MSL, respectively) in southern Poland. Five study sites along the OLK pollution gradient were established in an earlier study (Stefanowicz et al. 2008), and five study sites along the MSL gradient were established in the present study based upon metal concentrations in the soil organic layer (Table 1). The same unpolluted site was used for both gradients but was labeled by different metal concentrations in the soil organic layer; metal concentrations measured by Stefanowicz et al. (2008) were used for the OLK gradient unpolluted site, and metal concentrations measured by Tarasek (2011) were used for the MSL unpolluted site. Five samples of the whole soil organic layer were randomly taken from an area of approximately 100 m^2^ at each site on the OLK gradient, sieved (mesh size, 10 mm), and mixed (Stefanowicz et al. 2008). Ten samples of the whole soil organic layer from each site along the MSL gradient were taken while trapping the ground beetles and analyzed as described by Stefanowicz et al. (2008) except that each sample was analyzed individually for chemical characteristics. The contamination levels at all sampling sites are reported as total zinc and cadmium concentrations in the soil organic layer, as well as a pollution index (see below). All sites at both pollution gradients were located in mixed Scots pine, *Pinus sylvestris* L., forests on sandy podsolized soils with a small admixture of other species, including oak and birch.

**Table 1:** Concentrations of metals in soil organic layer and in the ground beetles *P. oblongopuncatus* (mean ± SD) and calculated pollution indices (PI) for beetles collected along two metal pollution gradients

Transect	Location	Distance from nearest smelter (km)	Metals in soil organic layer^a^			Metals in beetles (number of beetles analyzed in brackets)	
			Cd (mg/kg dry wt)	Zn (mg/kg dry wt)	PI	Cd (mg/kg dry wt)	Zn (mg/kg dry wt)
OLK	50°18′ N, 19°29′ E	3.9	39.1	1 763	21.3	1.6 ± 1.11 (24)	107 ± 15 (23)
OLK	50°19′ N, 19°30′ E	5.3	14.7	1 253	10.7	1.6 ± 0.97 (24)	103 ± 10 (23)
OLK	50°19′ N, 19°32′ E	7.9	12.2	755	7.6	1.8 ± 1.10 (24)	104 ± 15 (24)
OLK	50°25′ N, 19°38′ E	19.6	4.03	224	2.4	0.6 ± 0.48 (23)	99 ± 90 (24)
Unpolluted site 2008	50°32′ N, 19°39′ E	31.8	1.48	109	1.0	0.7 ± 0.65 (23)	100 ± 11 (24)
MSL	50°29′ N, 18°57′ E	2.1	52±10.6	2 684±689	20.5	3.7 ± 1.89 (32)	104 ± 16 (32)
MSL	50°31′ N, 18°56′ E	2.6	55±12.1	2 906±726	21.9	3.4 ± 1.41 (38)	106 ± 17 (42)
MSL	50°31′ N, 18°57′ E	3.3	36±10.1	1 886±520	14.3	2.3 ± 1.19 (51)	101 ± 15 (54)
MSL	50°34′ N, 19°58′ E	8.7	4.8±2.3	319±80	2.1	0.9 ± 0.44 (35)	100 ± 15 (36)
Unpolluted site 2010	50°32′ N, 19°39′ E	31.8	2.2±1.8	155±28	1.0	0.4 ± 0.37 (41)	95 ± 14 (43)

Pollution indices were calculated according to the equation ${\text{PI}} = \frac{{{\text{Z}}{{\text{n}}_i}/{\text{Z}}{{\text{n}}_{\text{U}}} + {\text{C}}{{\text{d}}_i}/{\text{C}}{{\text{d}}_{\text{U}}}}}{2}$, where index *i* denotes site number, index_*U*_ unpolluted site, and metal symbols stand for their concentrations in soil organic layer (in milligrams per kilogram dry weight)

*OLK* Olkusz, *MSL* Miasteczko Śląskie

^a^Data for OLK gradient from Stefanowicz et al. (2008), no SD indicated as chemical analysis were performed on mixed samples

Beetles were sampled with pitfall traps (approximately 200 ml capacity). Ninety traps were distributed per site in three lines of 30 cups each and emptied every second or third day between April 24 and May 15, 2008 (OLK) or in the same period in 2010 (MSL). The beetles were transported to the laboratory in plastic boxes with perforated lids and filled with litter from the collection sites. The beetles were separated by gender, placed individually in 30-ml plastic vials, and kept in a climatic walk-in chamber (20 °C, 60 % relative humidity, 16:8 light/dark photoperiod) for 24 h to void gut contents. The beetles were then weighed to the nearest 0.0001 g on an electronic balance (AS 160/C/2 Radwag, Poland), and at least 23 males from each site were frozen at −20 °C for metals analysis. The beetles used for biochemical analysis (ten males per site) were frozen in liquid nitrogen and stored at −80 °C.

### Energy reserves analysis

Legs, elytra, and wings were carefully removed from each beetle using forceps and a scalpel. The remaining body parts of each individual organism were used for biochemical analyses. All samples were homogenized on ice using a PRO 200 mechanical homogenizer (Bioeko, Poland), and measurements were performed on 96-well plates (Sarstedt, USA) using the μQuant spectrometer (Bio-TEK Instruments, USA). Each sample was analyzed twice or in triplicate.

The samples were homogenized on ice in 600 μl of ice-cold distilled water and the homogenate used for total lipid, carbohydrate, and protein measurements (adapted from De Coen and Janssen 1997). To measure lipid content, 250 μl chloroform, 250 μl methanol, and 125 μl Molecular Quality water (Sigma, USA) were added to 100 μl of homogenate. After centrifugation (1,000 × *g*, 5 min, 20 °C), the top phase was removed and 500 μl H_2_SO_4_ (98 %, POCH, Poland) added to 100 μl of lipid extract. After mixing, the sample was charred at 200 °C for 15 min, then cooled to room temperature, and 1.5 ml of distilled water added. The total lipid content was determined by measuring the absorbance at 400 nm using triglyceride tripalmitin (Sigma, USA) as a standard.

To determine the total protein and carbohydrate content, 100 μl of 15 % trichloroacetic acid (TCA) was added to 300 μl of homogenate and incubated at −20 °C for 10 min. After centrifugation at 1,000 × *g* for 10 min at 4 °C, the supernatant was collected and the pellet washed with 100 μl of 5 % TCA, centrifuged again, and the supernatant fraction combined with the previous fraction for use in the total carbohydrate analysis. The remaining pellet was re-suspended in 500 μl NaOH, incubated at 60 °C for 30 min, and neutralized with 300 μl HCl. The total protein content was assessed using Bradford’s reagent (Sigma, USA; Bradford 1976). The absorbance was measured at 592 nm using bovine serum albumin (Sigma, USA) as a standard. To determine the carbohydrate content of the supernatant fraction, 50 μl 5 % phenol and 200 μl H_2_SO_4_ was added to 50 μl of supernatant and quantification performed by measuring absorbance at 492 nm against a standard curve of glucose (Sigma, USA); since glycogen is hydrolyzed to glucose in hot sulfuric acid, this methodology can be used to determine also glycogen (Kemp and Kits van Heijningen 1953). The different energy reserves for each individual were converted into energetic equivalents using the energy of combustion (Gnaiger 1983) and summed: 17.5 kJ/g glycogen, 24 kJ/g protein, and 39.5 kJ/g lipids.

### Metal analysis

Whole beetles were dried at 105 °C for 24 h to obtain the dry mass, weighed to the nearest 0.0001 g, digested in 2 ml nitric acid (65 % Suprapur HNO_3_, Merck), and diluted to 5 ml with deionized water. Concentrations of zinc were analyzed by flame atomic absorption spectrometry (AAS, detection limit 0.011 mg/l), and cadmium was analyzed by graphite furnace AAS (detection limit 0.024 μg/l) (AAanalyst 800, Perkin-Elmer, USA). Three blanks and three reference samples **(**Certified Reference Material No 185—bovine liver (IRMM, Belgium) and Standard Reference Material No 1577c—bovine liver (NIST, USA), for OLK and MSL samples, respectively) were run to check the analytical precision. The measured concentrations were within ±5 % or −30 % of the certified reference value, for Zn and Cd, respectively. Metal concentrations in beetles were expressed as milligram per kilogram of dry mass.

### Statistical analysis

Prior to statistical analysis, extreme values with modified median absolute deviation scores greater than 3.5 were excluded and the data tested for deviation from normality using the Kolmogorov-Smirnov test. When this condition was not met, statistical analyses were performed on log-transformed data.

The total metal concentrations in soil organic layer were used for statistical analysis as the total concentrations of Zn and Cd were highly correlated with their water-soluble fractions (*r* = 0.97, *p* < 0.0001 and *r* = 0.98, *p* < 0.0001, respectively; Person correlation analysis). Correlations between Zn and Cd concentrations in the soil and beetles were checked using Pearson correlation analysis. Because the levels of metals in the soil highly correlated with each other (*r* = 0.99, *p* < 0.0001) and with body concentrations (*r* = 0.82 to 0.94, *p* ≤ 0.003), the study sites were described with a single measure of pollution defined as a *pollution index* (PI):

$$ {\text{PI}} = \frac{{{\text{Z}}{{\text{n}}_i}/{\text{Z}}{{\text{n}}_{{U}}} + {\text{C}}{{\text{d}}_i}/{\text{C}}{{\text{d}}_{{U}}}}}{2} $$

where *i* denotes site number, _*U*_ - the unpolluted site, and metal symbols denote their concentrations in soil.

Different metal concentrations for the same unpolluted site in 2008 and 2010 were used to take into account possible differences in soil sampling and metal analysis performed by different people. Simple regression analysis was performed to verify the effect of soil Cd and Zn pollution on body Cd and Zn concentrations.

Multiple linear regression analysis was used to select the variables that significantly affect total energy reserves and each component of the reserves (proteins, carbohydrates, and lipids) separately. The analysis was performed using average values from each study site as the concentrations of Cd and Zn could not be measured in the same animals in which energy reserves were assessed due to technical reasons. The independent variables in the model were the PI and average body mass. Average body masses were calculated for the beetles used in metal analyses and the beetles used in energy reserve analyses for each site. Body mass was incorporated into the model because earlier one-way analysis of variance revealed significant differences between sites in regard to the body mass of beetles used for metal analysis (*p* < 0.002). Variables with *F* > 4.0 were removed from the model (backward stepwise procedure). The percentage of total variance explained by the final regression models was expressed as *r* ^2^.

Because the beetles were collected from the same unpolluted site in 2008 and 2010, possible differences in energy reserve components between different years due to factors other than metal pollution were tested for using *t* test. All statistical analyses were performed with Statgraphics Centurion XVI (StatPoint Technologies, Inc., USA).

## Results

Among 120 measures of body Zn or Cd concentration at the OLK gradient, two values for Cd and two values for Zn were excluded from statistical analysis as outliers. For MSL (*n* = 207), ten outliers for body Cd concentration were excluded. Among 98 beetles analyzed for a particular component of energy reserves, one outlier was excluded from protein analysis and two from carbohydrate analysis, all from the MSL gradient. For technical reasons, the lipid contents were measured in 95 samples, and all samples were taken into account in further statistical analysis.

**Fig. 1:**
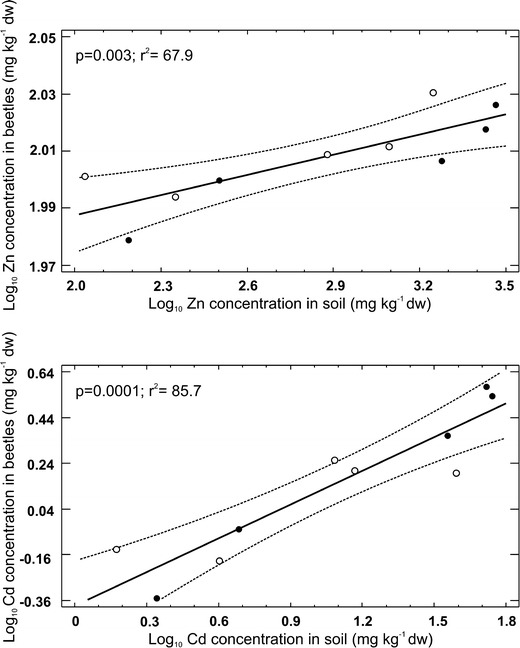
Effect of metal concentration in soil on the internal body concentration of metals in *P. oblongopunctatus* collected along two pollution gradients. *Upper panel*, Zn; *lower panel*, Cd; *open circles*, OLK; *full circles*, MSL

Both gradients covered a broad range of contamination levels, from virtually uncontaminated to highly contaminated with a mixture of metals. The highest concentrations of Zn and Cd were found near the Miateczko Śląskie smelter (up to 2,906 and 55 mg/kg, respectively, compared to the concentrations at the unpolluted site of 155 mg Zn/kg and 2.2 mg Cd/kg). The highest body concentrations of Zn were found in beetles collected from the Olkusz gradient (107 mg/kg dry bw), whereas beetles collected near Miasteczko Śląskie smelter accumulated the highest concentrations of Cd (3.7 mg/kg dry bw). Detailed data on the metal concentrations in the soils and beetles are given in Table 1. Both body Zn and Cd concentrations increased with increasing soil Zn and Cd concentrations (*p* = 0.003, *r* ^2^ = 67.9 and *p* = 0.0001, *r* ^2^ = 85.7, respectively; Fig. 1). Although the relationship for Zn was significant, the range of average body Zn concentrations observed along contamination gradients was very narrow (95–107 mg/kg dry bw), indicating that the beetles are able to regulate Zn efficiently.

All energy reserve components exhibited large variation. Proteins and lipids were quantitatively the most important energy fractions in the biochemical composition of *P. oblongopunctatus* (992 to 1,748 J/g wet wt and 716 to 2,210 J/g wet wt, respectively), whereas carbohydrates represented only 22 to 97 J/g wet wt (Table 2).

**Table 2:** Energy reserves (in joules per gram wet weight) in proteins, carbohydrates, and lipids and total energy reserves in the ground beetles *P. oblongopuncatus* (mean ± SD) collected along two metal pollution gradients

Transect	Distance from nearest smelter (km)	Proteins	Carbohydrates	Lipids	Total energy reserves
OLK	3.9	1,644 ± 386 (9)^a^	29 ± 15.8 (9)	1,655 ± 848 (8)	3,308 ± 1,122 (8)
OLK	5.3	1,748 ± 288 (10)	37 ± 14.3 (10)	1,728 ± 1,010 (9)	3,485 ± 1,056 (9)
OLK	7.9	1,719 ± 347 (10)	22 ± 8.30 (10)	1,776 ± 782 (10)	3,517 ± 910 (10)
OLK	19.6	1,480 ± 270 (10)	31 ± 5.62 (10)	2,210 ± 1,461 (10)	3,720 ± 1,587 (10)
OLK	31.8	1,522 ± 561 (10)	44 ± 29.4 (10)	1,271 ± 523 (10)	2,870 ± 1,134 (9)
MSL	2.1	1,218 ± 332 (10)	92 ± 36.2 (10)	817 ± 536 (10)	2,128 ± 695 (10)
MSL	2.6	1,165 ± 317 (10)	97 ± 30.2 (10)	752 ± 368 (10)	2,015 ± 559 (10)
MSL	3.3	992 ± 231 (9)	97 ± 20.6 (10)	1,075 ± 856 (9)	2,249 ± 857 (9)
MSL	8.7	1,099 ± 350 (10)	92 ± 24.1 (9)	716 ± 554 (10)	1,751 ± 611 (9)
MSL	31.8	1,236 ± 390 (9)	79 ± 23.7 (8)	972 ± 537 (8)	2,281 ± 656 (7)

*OLK* Olkusz, *MSL* Miasteczko Śląskie

^a^Number of beetles analyzed

**Fig. 2:**
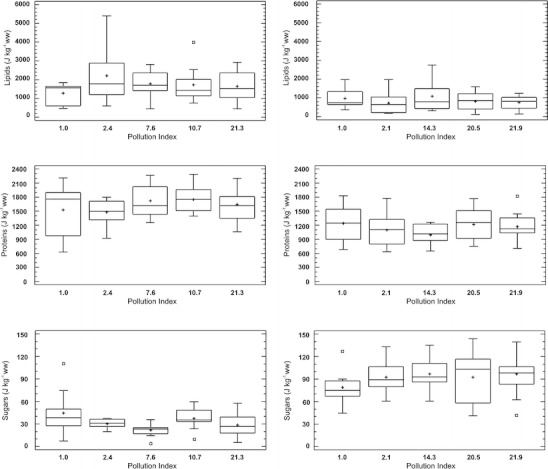
Box-and-whisker plots for energy reserves (proteins, lipids, and carbohydrates) in *P. oblongopunctatus* collected from sites with different pollution indices at the OLK (*left hand column*) and MSL gradients (*right hand column*). The *plus sign* on each box represents the mean value, the *center line* shows the sample median, the *boxes* contain lower and upper quartiles, and the *whiskers* extend to the minimum and maximum values. The most extreme values outside this range are shown as *small squares*

Neither the energy reserves nor any of the energy reserve components (lipids, carbohydrates, and proteins) depended on the PI (Fig. 2). The only significant relationship was the increase in lipid fraction with body mass; the heavier beetles had more lipids per unit of body mass (*p* = 0.045, *r* ^2^ = 41.3 %). A significant difference was measured in the carbohydrates concentration between the means of the two unpolluted; beetles collected in 2010 had higher carbohydrate levels than those collected in 2008 (*p* = 0.016) (Fig. 2).

## Discussion

With increasing levels of stress, the maintenance of physiological integrity becomes more difficult for organisms. Although energy-demanding homeostatic processes can initially provide comfort, they will eventually break down as soon as energy reserves become depleted (cf. Calow 1991; De Coen and Janssen 2003a), initiating a series of adverse effects. Thus, energy reserves provide valuable information about the capacity of an organism to cope with stress long before adverse effects are manifested at higher levels of biological complexity (Smolders et al. 2003). In this context, the energy status of *P. oblongopunctatus* was studied to gain insight into the costs of living in an environment with metal pollution. To enable strong and more general inference of the influence of metal pollution on energy reserves, and to exclude, as much as possible, other factors in the natural environment as a reason for possible differences in energy reserves between study sites, the research was carried out at two metal pollution gradients. The earlier study on closely related species *Pterostichus melanarius* indicated no correlation between sampling time and the percent of fat in beetles collected between April 1 and May 31 (Lindquist and Block 2001). However, at least some of the females were likely already fertilized (Brunsting 1981), and this is known to influence metabolism (Chaabane et al. 1999). Therefore, to avoid a large variation caused by sex-specific metabolism, as well as sex-specific differences in the accumulation of metals (Butovsky 2011), only males were used in this study.

No significant relationships between total energy reserves or the levels of their particular components and increasing metal concentration in the soil or beetles’ bodies were found in this study. All biochemical fractions (proteins, carbohydrates, and lipids) exhibited large variation. A similarly large variation was observed in field-collected estuarine mysid *Neomysis integer* (Verslycke et al. 2004b). According to De Coen and Janssen (1997), each species has an individual available energy reserve distribution, and in the case of *Daphnia magna*, non-protein substrates are the preferred energy source*.* This study suggests that the main storage materials in *P. oblongopunctatus* are proteins and lipids, whereas carbohydrates were present at a relatively low level. The synthesis and utilization of energy reserves (i.e., fat and glycogen) are controlled in insects by fat body cells (Arrese and Soulages 2010). Much higher lipogenesis from glucose compared to glycogen synthesis explains the higher lipid content compared to glycogen in the insect body (Arrese and Soulages 2010) and may explain the high lipid content in *P. oblongopunctatus*. Glycogen is mobilized for the production of trehalose and sugar alcohols under suboptimal temperatures and drought and plays a role in preventing cellular damage at low temperatures and during diapause (Arrese and Soulages 2010). Studies of several insect species have shown that cold acclimation leads to an increase in the body content of trehalose and glucose (Storey 1990; Vanin et al. 2008). Therefore, the difference in carbohydrate concentration between beetles collected in 2008 and 2010 from unpolluted sites could be, at least partly, the consequence of a severe winter preceding sampling in 2010. Analysis of meteorological data from the Research Station of the Institute of Geography and Spatial Management in Gaik-Brzezowa (Wieliczka Foothills) revealed that the average daily minimum and maximum air temperature during winter (31st December–31st March) were lower in 2010 (−4.3 and 2.3 °C, respectively) compared to 2008 (−0.8 and 5.7 °C, respectively).

Considering the components of available energy reserves, proteins are indicated as a prominent element for building enzymes, hormones, and antibodies in organisms, whereas glycogen and lipid reserves are used preferably to proteins in response to stress (Smolders et al. 2003). However, different species can use different strategies to survive in heavily metal-contaminated areas, and various effects of metals on energy reserves in terrestrial invertebrates have been reported. Donker (1992) showed that the isopod *Porcelio scaber* collected from metal-polluted areas with remarkably high Zn and Cd concentrations in their bodies have reduced energy reserves (i.e., protein, glycogen, and lipid content). On the other hand, neither glycogen nor total protein content in *P. scaber* collected from contaminated or uncontaminated sites were diminished by exposure to lead in the laboratory (Knigge and Köhler 2000). Schill and Köhler (2004) showed that the glycogen and lipid content of midgut gland cells from *P. scaber* did not depend on the distance from the pollution source in animals collected from long-term polluted sites in the vicinity of a lead–zinc smelter in Avonmouth, UK. Also, Holmstrup et al. (2011) did not find any correlation between internal Cd or Pb concentrations and glycogen reserves in earthworms (*Dendrobaena octaedra*), despite high body concentrations of metals. In contrast to the metals occurring at high internal concentrations, metals that are regulated by earthworms and kept at low levels (Ni, Al, and in one case, also Zn) caused a reduction in glycogen reserves. The authors concluded that the detoxification of metals by storage in granules as inert precipitates, as in the case of Pb and Cd, seems to be associated with decreased energy demands compared to active regulation of internal concentrations (Ni, Al) by the worms. Undoubtedly, also beetles are able to regulate concentrations of nutritional metals more efficiently than those of xenobiotic metals (Kramarz 1999; this study). The storage of metals as an energy efficient method of detoxification and subsequent excretion via feces was earlier suggested by Simkiss (1976, 1977).

To verify that metal pollution has an effect on the energy content of the body, Zygmunt et al. (2006) measured the body caloric value of *P. oblongopunctatus* but found no relationship with the level of metal contamination in the soil. Another study aimed at assessing the energy reserves of *Poecilus cupreus*, a carabid species closely related to the one studied in this paper, used the caloric value of the organism as an indicator of stress (Maryański et al. 2002). The authors showed a decrease in the body caloric value with increasing food contamination. However, the results were obtained in a laboratory experiment with laboratory-reared animals fed with artificially contaminated food and kept individually under optimal conditions; thus, any confounding factors, such as competition, were excluded. This observation may suggest that ecological interactions, such as decreased competition for resources at polluted sites, can counterbalance the costs of detoxification.

Different resistance mechanisms have different theoretical costs (Coustau et al. 2000), and the indirect evidence that carabids bear the costs of living in a polluted environment can be deduced from a decline in the fertility of female beetles from more contaminated sites (Łagisz et al. 2002) and lower quality of produced eggs (Lagisz and Laskowski 2008). Stone et al. (2001) showed that *P. oblongopunctatus* inhabiting chronically metal-polluted sites were less tolerant to additional environmental stressors (e.g., food deprivation or pesticide) than those from less contaminated areas. Further study of this species did not reveal the genetic basis of this increased susceptibility to additional stressors (Lagisz and Laskowski 2007). Metal resistance often involves an increased production of ligands, proteins, and enzymes involved in detoxification, which must bear some costs. Therefore, a reduction in an animal’s energy reserves or increases in their metabolic rates have been associated with metal toxicity (Moolman et al. 2007). Although many short-term studies on aquatic invertebrates have shown that energy reserves are sensitive endpoints that respond rapidly to chemical stressors (De Coen and Janssen 2003a; Smolders et al. 2004), the depletion of energy reserves was seldom found in terrestrial invertebrates exposed in situ (Donker 1992).

## Conclusion

*P. oblongopunctatus* are able to survive in metal-polluted environments without any serious impact on their energy reserves, though they obviously have to cope with high body metal concentrations. The energy reserves of the studied carabids were remarkably stable, independent of the pollution. The mechanisms that endow the *P. oblongopunctatus* from metal-polluted areas with resistance to metal toxicity remain unclear.
